# Neuro-Inflammation Modulation and Post-Traumatic Brain Injury Lesions: From Bench to Bed-Side

**DOI:** 10.3390/ijms231911193

**Published:** 2022-09-23

**Authors:** Alice Jacquens, Edward J. Needham, Elisa R. Zanier, Vincent Degos, Pierre Gressens, David Menon

**Affiliations:** 1 Unité de Neuroanesthésie-Réanimation, Hôpital de la Pitié Salpêtrière 43-87, Boulevard de l’Hôpital, F-75013 Paris, France; 2 Inserm, Maladies Neurodéveloppementales et Neurovasculaires, Université Paris Cité, F-75019 Paris, France; 3 Division of Anaesthesia, Addenbrooke’s Hospital, University of Cambridge, Box 93, Hills Road, Cambridge CB2 2QQ, UK; 4 Department of Neuroscience, Istituto di Ricerche Farmacologiche Mario Negri IRCCS, 20156 Milan, Italy

**Keywords:** traumatic brain injury, neuroinflammation, neuroprotection, therapeutics

## Abstract

Head trauma is the most common cause of disability in young adults. Known as a silent epidemic, it can cause a mosaic of symptoms, whether neurological (sensory–motor deficits), psychiatric (depressive and anxiety symptoms), or somatic (vertigo, tinnitus, phosphenes). Furthermore, cranial trauma (CT) in children presents several particularities in terms of epidemiology, mechanism, and physiopathology—notably linked to the attack of an immature organ. As in adults, head trauma in children can have lifelong repercussions and can cause social and family isolation, difficulties at school, and, later, socio-professional adversity. Improving management of the pre-hospital and rehabilitation course of these patients reduces secondary morbidity and mortality, but often not without long-term disability. One hypothesized contributor to this process is chronic neuroinflammation, which could accompany primary lesions and facilitate their development into tertiary lesions. Neuroinflammation is a complex process involving different actors such as glial cells (astrocytes, microglia, oligodendrocytes), the permeability of the blood–brain barrier, excitotoxicity, production of oxygen derivatives, cytokine release, tissue damage, and neuronal death. Several studies have investigated the effect of various treatments on the neuroinflammatory response in traumatic brain injury in vitro and in animal and human models. The aim of this review is to examine the various anti-inflammatory therapies that have been implemented.

## 1. Introduction

Traumatic brain injury (TBI) is defined as an alteration in brain function caused by an external force [1]. However, disagreements persist regarding this definition, which may account for the literature’s heterogeneity regarding its epidemiology [2,3,4]. TBI is one of the main causes of death and disability in young people, and its incidence is estimated at 69 million per year worldwide [5]. Currently, the incidence of TBI in Europe is estimated at 9.3 million annual cases, increased from 3.7 million in 2004. In France, this figure is around 150 cases per 100.000 inhabitants [6] but it is probably underestimated because not all patients coming to the emergency room are hospitalized [7]. Also in France, the overall incidence of severe TBI has been steadily decreasing for thirty years: 24/100,000 in 1986, 17/100,000 in 1996 and 3/100,000 in 2007 [8]. This decrease is accounted for primarily by patients under 55 years of age; conversely, there is an increase in the incidence of TBI in people over 75 years of age [9]. TBI severity is typically stratified by Glasgow Coma Scale (GCS) on admission, and in turn GCS has a role in predicting outcome, although much smaller than might be expected, suggesting that factors other than the initial injury severity are important determinants in outcome, and may be therapeutic targets. [10,11]. 

A severe TBI should never be assumed to be isolated; indeed, two descriptive studies demonstrate the ubiquity of associated injuries: 70% with fractures of the extremities, 35% thoracic trauma, 20% abdominal trauma, and 8% cervical spine trauma [12,13]. The neuroinflammatory response to TBI therefore occurs in the context of significant peripheral inflammation, which can in turn exacerbate neuroinflammation [14]. All studies agree that the risk of TBI is higher in men regardless of age. The incidence of TBI varies by age, with a trimodal distribution: young children (0–4 years), adolescents and young adults (15–24 years) and the elderly (>65 years). Many studies have looked at the causes of TBI. It seems evident that the aetiologies depend on sex and age groups and are also correlated with the severity of the trauma. Some studies have focused on a certain type of cause other than TBI associated with sport or military activities. The injury mechanisms are mainly linked to falls (extreme ages) and road traffic accidents (RTA) [15]. Alcohol intake and/or the use of illicit substances is found in 60% of severe TBI.

**Figure 1:**
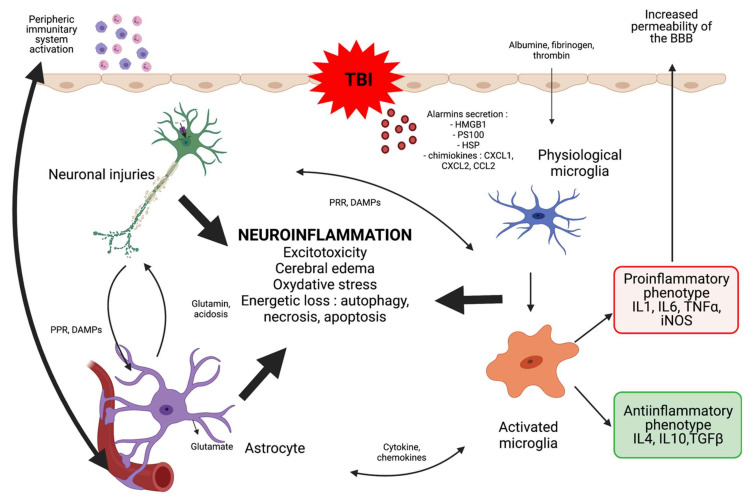
Schematic representation of post-traumatic neuroinflammation. The rupture of the blood-brain barrier, the release of alarmines (or DAMPS: Damage Associated Molecular Patterns) by injured cells and the production of cytokines are at the origin of an endothelial, astrocytic, and microglial activation with a change in conformation of the microglial cells which take an amoeboid conformation and migrate towards the injured area. This response to trauma is both localized and generalized with secondary recruitment of the peripheral immune system. BBB: blood brain barrier; CXCL: chemokine ligand; DAMPS: damaged associated molecular patterns; IL: interleukin; HMGB1: high mobility group bow 1; iNOS: inducible nitric oxide synthase; PRR: pattern recognition receptor; PS100: protein S 100; TBI: traumatic brain injury; TGF: transforming growth factor; TNF: tumor necrosis factor.

The pathophysiology of TBI is complex and, in addition to the damage resulting from direct physical injury, pressure-effects and ischaemia, involves a constellation of different intra- or extra-cerebral immunological cellular actors interacting via multiple small molecules (cytokines, or chemokines) through a permeabilized blood-brain barrier (BBB) thus generating an inflammatory storm shown schematically in Figure 1. Historically, two types of post-traumatic injuries have been described: immediate primary injuries related to the impact itself, and secondary injuries that appear within hours and months of trauma. It is only recently that a third type of lesion has been discussed: the so-called late tertiary lesions. Primary lesions result directly from the impact, and in particular from what is classically called “container-content conflict”. There are three physical mechanisms: direct contact trauma, the most frequent; the phenomena of acceleration or deceleration called inertia effect, responsible for disseminated lesions; and finally the static compressive mechanism called crushing, the rarest. The resulting lesions can be focal and multifocal. The focal are mainly represented by cortico-subcortical cerebral contusions, haemorrhages of venous origin most often resulting from the direct impact of the brain against the skull, and different variations of hematoma: epidural (EDH), subdural (SDH), intraparenchymal (IP), or subarachnoid hemorrhage (SAH). These lesions are often very well visualized in the acute phase on computed tomography (CT) scan. Contusions are present under the impact zone (direct contusion by blow) or at a distance (indirect contusion by backlash), and most typically occur in the frontal and temporal lobes. The multifocal or diffuse lesion subtype is also known as diffuse axonal injury (DAI)—little or not at all detectable with conventional neuroradiology techniques, but visualizable in post-mortem histopathology. They are mainly linked to acceleration-deceleration movements leading to shearing and stretching effects, and are found mainly in areas of lower resistance of axons (transition areas between gray matter and white matter) [16], especially in the corpus callosum, the subcortical white matter, and the brainstem [17]. These lesions are frequent in cases of high kinetic trauma in young subjects. In 2010, Skandsen et al. observed DAI in 72% of patients with moderate to severe TBI, associated in 50% of cases with focal lesions. They are a major cause of persistent coma and vegetative state [18,19] and in survivors they are responsible of significant functional and motor sequelae [20].

Secondary lesions can appear within the first few minutes after the TBI and will worsen the initial lesions. They develop in areas of cerebral parenchyma still viable but weakened by primary cerebral insult, and are mediated by factors of systemic or central origin, usually concomitantly. Arterial hypotension, hypoxia, hypercapnia, anemia, as well as some metabolic and fluid electrolyte disturbances are all systemic factors contributing to the development of secondary lesions [21,22,23]. At the cellular level, all of these secondary attacks cause neuronal death via multiple mechanisms with an inflammatory storm linked to both a peripheral and local immune reaction in the central nervous system (CNS) [24,25,26,27]. This inflammatory reaction breaks down into two parts: first, a central intracerebral inflammatory response with in particular glial activation, then an added systemic immune response. 

TBI induces immediate neuropathological effects which may be transient in the less severe forms. However, with increasing severity, it exacerbates neuronal damage by degeneration mechanisms. The latter, described in the literature, may occur remotely from the acute phase. The pathophysiological mechanisms of these so-called tertiary lesions are still uncertain but would involve processes common to certain dementias and inflammatory diseases of the CNS leading to neurodegeneration. The two main pathological arguments in favor of this analogy are cerebral atrophy and cerebral inflammation: a postmortem study conducted by Johnson et al. demonstrated a significant decrease in the thickness of the corpus callosum in 22 subjects who had been victims of a TBI more than a year ago, compared to control subjects; microglial reactivity is also observed in 30% of patients after one year following TBI, indicating persistent brain inflammation [28].

**Figure 2:**
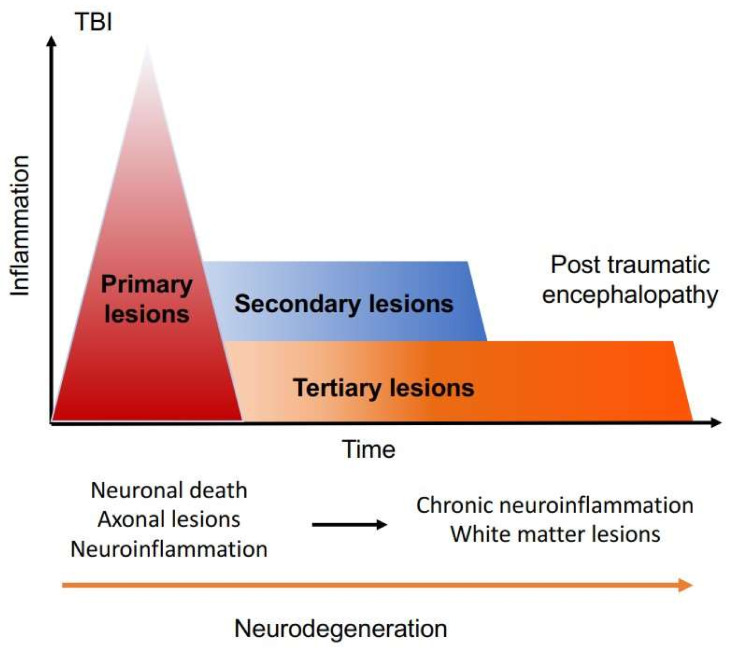
Chronology of post-traumatic lesions. The TBI is responsible for a primary mechanical lesion that can be transformed into a tertiary lesion via neuro-inflammatory mechanisms and thus generate a chronic post-traumatic encephalopathy. TBI: traumatic brain injury.

Tertiary lesions are thought to influence the long-term prognosis and constitute a clinical entity called chronic post-traumatic encephalopathy (Figure 2).

Severe TBI has long been considered an exclusively acute clinical entity, and the notion of new brain lesions occurring long after the initial impact was not introduced until the 1920s [29]. This hypothesis was already mentioned at the time but it was probably experienced as an inevitability, but over the last thirty years, several studies have focused on better understanding the mechanisms involved in these lesions in order to identify therapeutic targets. 

To date, the phenomenon previously stated as pugilistic dementia has been redefined as post-traumatic encephalopathy (PTE), a pathology that has been widely studied [30]. Historically, it was initially described in high performance athletes [31,32,33,34,35,36] but it is now known that it can develop in any patient who has undergone a single TBI. PTE can result in disorders of attention, memory, and concentration with significant impact on patient social functioning and quality of life. PTE can progress to Alzheimer-like dementia in the decades following the trauma. Anatomically, it is characterized by the deposition of hyperphosphorylated TAU protein in neurofibrillary tangles, most often in the perivascular spaces, the depths of the cortical grooves, and the subpial and periventricular areas.

Recently, a new nosological entity called post-concussion syndrome (PCS) has been described [37]. Historically, this entity has been confused with post-traumatic stress disorder (PTSD), though it has clear clinical distinctions. Indeed, complaints reported by TBI patients include not only a constellation of psychiatric symptoms that may overlap with PTSD, such as anxiety, aggressiveness, emotional lability, sleep and eating disorders, memory and attention disorders, and difficulty concentrating, but also somatic symptoms such as headache, dizziness with tinnitus or phosphenes. The Paris-TBI study, which examined the long term outcome of TBI patients treated in Ile-de-France between 2005 and 2007, showed that between 1 and 4 years after the TBI, 39% of the 147 patients had improved clinically while 43% remained stable and 15% had worsened. Thus at 4 years of the trauma only 28% had recovered, while 40% suffered moderate handicap and 32% severe handicap [38]. At present, the literature does not explain why some patients will develop PCS and others will not, or why some worsen later. Even if some genetic susceptibilities may exist, neuroinflammation could be one of the main explanations for this clinical expression.

In addition, the clinical course after TBI does not appear to be linear. Indeed, some studies observe a rapid improvement in the first months, followed by a slower progression and then a plateau phase with, in the most severe cases, an absence of return to the premorbid state [39,40,41]. TBI in children, apart from anatomical differences, also has some clinical specificities with a particularly serious impact in the long term.

The management of the acute phase of head trauma is currently the subject of multiple medical recommendations with both multimodal monitoring tools (intracranial pressure, microdialysis) and therapies to control secondary lesions and more specifically intracranial hypertension (osmotherapies, coma, hypothermia, even surgical treatment) [42,43]. These measures significantly reduce the acute morbidity and mortality linked to head trauma, but unfortunately do not improve post-traumatic disability. The latter results from the accumulation of insults in the acute phase and particularly from neuroinflammation. The main objective below is to present and discuss the various neuro-antiinflammatory treatments that have been studied in the literature over the years (Table 1).

## 2. Neuro-Anti-Inflammatory Therapeutics

In this first part, we discussed anti-inflammatory therapeutics such as glucocorticoids and non-steroidal anti-inflammatory drugs and also drugs such as melatonin, cyclosporine, oxytocin, statins, and erythropoietin, whose initial mechanism of action is not anti-inflammatory but which can, in certain contexts—similar to TBI—have an anti-inflammatory activity. 

### 2.1. Glucocorticoids

Glucocorticoids (GC) havethe ability to act on various neuroinflammatory mechanisms. They are also known to reduce vasogenic edema but on the other hand worsen cytotoxic edema [44]. In addition, GCs have the potential to act on all three phases of post-traumatic injury (TBI), so their use in TBI seems particularly attractive. Derived from the natural hormone cortisol, synthetic GCs are drugs developed to maximize glucocorticoid effects and minimize mineralocorticoid effects. Synthetic derivatives that have appeared on the market vary in anti-inflammatory efficacy, half-life, and mineralocorticoid action, but all have structural similarity and a common mechanism of action: they circulate bound to transport proteins, with a small fraction of pharmacologically-active unbound form. This free fraction crosses the cell membrane and binds, with high affinity, to specific cytosolic receptors called nuclear glucocorticoid receptors (GR) which can then enter the nucleus. 

GCs are steroidal anti-inflammatory drugs. Cortisol, also called hydrocortisone, has glucocorticoid properties (particularly anti-inflammatory) and mineralocorticoid properties (anti-diuretic, anti-natriuretic, and kaliuretic). Their main property is immunomodulation. First, corticosteroids reduce the synthesis of many pro-inflammatory cytokines and chemokines (TNF-α, IL-1ß, IL-2, IL-6, IL-8, IL-4, IL-5, IL-12, IL-17, IL-18, GM-CSF…). They also induce the synthesis of anti-inflammatory lymphocyte cytokines such as IL-10 and TGF-ß [45].

In addition, corticosteroids directly inhibit the synthesis of multiple inflammatory enzymes, such as inducible NO synthetase (iNOS), phospholipases A2 and C, but also cyclooxygenase 2 [3,4]. Many other enzymes involved in the phenomena of cell destruction (proteases, collagenases) or in inflammatory phenomena (C3 convertase) are also inhibited.

Corticosteroids also act on many target cells involved in innate or adaptive immunity [46]: macrophages, dendritic cells, polymorphonuclear cells, and T and B lymphocytes in particular.

They act by controlling their maturation, regulating their activation, modulating their capacity for synthesis (cytokines, chemokines, enzymes, etc.), managing their survival and migration, and by modifying their “learning”, in particular for intrathymic lymphocytes [47]. They can reduce release of lysosomal enzymes and preformed granules containing inflammation mediators (histamine, serotonin). This partly explains the inhibition of cellular activity seen with corticosteroids, especially for immune cells such as lymphocytes. Finally, the effectiveness of corticosteroids is directly related to the cytosolic concentration of the receptor of the GCs (RGCs) available. However, the affinity and cytosolic concentration of GRs are genetically regulated. A particular polymorphism of RGCs could explain an increased sensitivity to corticosteroids in some patients [48]. 

Finally, corticosteroids inhibit peroxidation and lipid hydrolysis. Effects on the maintenance of aerobic energy metabolism, intracellular accumulation of calcium, and the preservation of cerebral blood flow have also been attributed to them. Experimental data supports neuroprotective action of corticosteroids in models of TBI [49].

Corticosteroids have been used effectively for several years in inflammatory neurological conditions in humans. They are indicated in multiple sclerosis, oncology, and postoperative neurosurgery to reduce peritumoral edema [50]. Concerning TBI, a survey in England carried out in 1996 shows that these drugs were used in 14% of units with TBI patients [51]. Unfortunately, however, the literature is not definitive regarding their effectiveness: meta-analyses show that the work to date is too heterogeneous in terms of patient demographics, doses, corticosteroid type, and timing and duration of treatment [52,53,54,55].

### 2.2. Nonsteroidal Anti-Inflammatory Drugs 

Nonsteroidal anti-inflammatory drugs (NSAIDs) are non-selective COX and selective COX2 inhibitors. Tested on several models of TBI, they reduce neuroinflammation by inhibiting the production of IL-1ß, IL-6 and IL-10, but do not reduce tissue damage and functional consequences of TBI [56].

### 2.3. Statins

Statins are HMGCoA reductase (hydroxy-methyl-glutaryl-coenzyme A reductase) inhibitors and form a class of lipid-lowering drugs commonly used to control cardiovascular risk factors. In addition to this beneficial effect on lowering cholesterol, statins have an anti-inflammatory effect by reducing oxidative stress through up-regulation of endothelial NO-synthase. However, other signaling pathways also intervene in parallel with the NO-synthase one, such as tissue plasminogen activator and the phosphoinositide 3-kinase (PI3K)/serine-threonine kinase (AKT) pathway.

Statins appear to be a potentially interesting neuroprotective strategy, especially during stroke, subarachnoid hemorrhage, and possibly TBI [57,58,59]. Some authors also show that statin treatment likely not only promotes hippocampal neurogenesis in the dentate gyrus but also improves learning [60]; and possibly reduces apoptosis [59]. Statins can decrease apoptotic cell death and promote neuron survival by suppressing Caspase-3 activity [61,62,63] and by reducing the Bax/Bcl-2 ratio [60]. Furthermore, acute statin treatment attenuates microglial activation and polarization after TBI in rodents [61,64,65]. In addition to protecting pre-existing neurons, statins foster neurogenesis with growth and neuronal differentiation particularly in the hippocampus probably due to an upregulation of neurotrophic factors like brain- derived neurotrophic factors or vascular endothelial growth factor [60,66,67]. Statins also have vascular and endothelial effects. In a murine model of TBI, atorvastatin decreased the level of delayed thrombosis, and this was correlated with a reduction in necrotic brain tissue [68]. Multiple mechanisms are probably associated, but pre-clinical and clinical studies have demonstrated that statin treatment can decrease pro-thrombotic markers like von Willebrand factor [69]. Statins have also been shown to promote angiogenesis in TBI models, with an increase in newly formed vessels and capillary density and VEGF (Vascular Endothelial Growth Factor) levels [66]. The effect of statins could also be anti-inflammatory via decreased expression of pro-inflammatory markers such as TLR (Toll like receptor) 4, NFκB, and IL(interleukin)-1ß or IL-6 [70,71,72]. Statins can also lower microglial activation [64], also probably due to the effect of statin on harmful oxygen free radicals, such as superoxide production [73]. This decrease of pro-inflammatory markers is associated with blood brain barrier (BBB) integrity maintenance [72,74,75]. In a systematic review published in 2021, the authors showed four randomized clinical trials with 296 patients demonstrating that statins can play a neuroprotective role and improve cognitive outcomes by anti-inflammatory effect, for example, in association with lower tumor necrosis factor and c-reactive protein [76]. In summary, statins appear to be a good candidate for further studies on improving cognitive outcome after traumatic brain injury. 

### 2.4. Melatonin

Melatonin (N-acetyl-5methoxytryptamine) is a hormone produced by the pineal gland. It has many functions that are particularly interesting in the context of brain injury [77]. 

Recently Osier et al. published a review on published evidence of therapeutic mechanisms of melatonin in TBI [77]. It acts as a powerful antioxidant by promoting the elimination of reactive oxygen species (ROS) by trapping and reducing the synthesis of iNOS and nNOS while increasing that of antioxidant enzymes [78]. These antioxidant capacities are confirmed in models of cerebral ischemia, in which melatonin reduced the size of the ischemic lesion and generated an anti-inflammatory response [79,80,81,82]—findings also observed in the context of TBI [83,84,85]. Melatonin also appears to have mitochondrial protective properties by helping to maintain their structure and function [86,87]. One of the other hypothesis is that melatonin could overcome energy depletion via the adenosine 3′5′monophosphate (AMP)-c/p- cAMPc-response element binding (CREB) pathway; in this worked melatonin treatment also decreased apoptotic cell death, lesion volume, and promote post-TBI motor coordination and work memory [88]. Furthermore, melatonin was reported to reduce neuroinflammation and brain edema, decrease late-phase activation of NFkB [89] and attenuate acute microglial and astrocyte activation [90]. 

Clinically, studies have shown mixed and conflicting results on the effectiveness of melatonin for the treatment of TBI. In pediatric populations, melatonin appears to demonstrate neuroprotective effects [91]: PLAYGAME, a randomized controlled trial, tested different doses of melatonin on children ages 13 to 18 with post-concussion syndrome symptoms [92]: in addition to anti-neuroinflammatory effects, melatonin seems to modulate neurobehavioral and particularly sleep cycle difficulties, and indirectly cognition. However, in a double-blind randomized crossover trial with post-TBI adults suffering from sleep disorders, melatonin supplementation did not improve patients’ sleep or neuropsychiatric well-being [93]. In contrast, another randomized double blind placebo-controlled study found melatonin supplementation over a 4-week period safely and effectively improved subjective sleep quality evaluated by the global Pittsburgh Sleep Quality Index score [94].

Melatonin appears to be a potentially useful anti-inflammatory agent in TBI, particularly due to its anti-oxidant effects. Its absence of toxicity allows for clinical studies. Clinical trials are currently discordant but it could be that melatonin can act on post-TBI sleep disorders. The effects of melatonin appear to be through indirect mechanisms but some studies have looked at other mechanisms that could be specific to melatonin and its receptors [95]. 

### 2.5. Minocycline

Minocycline is an antibiotic from the cycline family, the second-generation tetracyclines, and has been demonstrated to protect against neonatal hypoxic-ischemic injury in a rodent model [96]. It also exhibits numerous neuroprotective effects in different animal models of TBI, such as inhibition of microglial activation [97,98] and reduction of cytokine production, including IL-1β and IL-6 as well as chemokines CCL4, CXCL1 and CXCL2 [99]. It reduces the production of nitric oxide [100], and inhibits the excitotoxic N-methyl-D-aspartic acid pathway [101] and ROS, with an associated decrease in cerebral edema and lesion volume. 

In a mild blast-induced model of TBI, minocycline treatment normalized tissue levels of inflammatory (CRP), vascular, neuronal (Neuron Specific Enolase [NSE], tau), and glial markers (Glial fibrillary acidic protein [GFAP], S100B) [102]. Others studies have shown negligible effect, however, a 2018 study on neonatal rats found minocycline was ineffective in reducing microglial/macrophage activation and ameliorating post-injury deficit by postnatal day 11 [103]. In another study that similarly found no effect of minocycline, the authors tested two time points for treatment: 1 h or 9 weeks post-injury, with no effect on lesion size or degree of microglial activation after either the early or the late administration of minocycline [104]. 

Some studies show that minocycline plus N-acetylcysteine (NAC) synergistically improve cognition and memory, modulating neuroinflammation and preventing oligodendrocyte loss [105], preserving myelin, limiting lesion volume [106,107], and promoting remyelination [108]. However, these results seem not to apply in clinical practice as thus far, minocycline has not been shown to benefit neurological outcomes [109]. Recently a clinical study examined the effect of minocycline in the chronic phase of TBI: fifteen patients received either minocycline or *placebo* at least 6 months after TBI, and the authors observed that while minocycline treatment reduced chronic microglial activation by PET, it increased plasma concentrations of neurofilament light, a marker of neurodegeneration [110]. In another clinical study—the phase IIa open label safety and feasibility study for preliminary data on functional outcomes – Meythaler et al. demonstrated a trend in neurologic improvement for the higher dose of minocycline, but this did not reach statistical significance. These two clinical studies are limited, however, in that they are underpowered. Further higher-powered studies may demonstrate clinical efficacy, but for the moment, minocycline has not been shown to improve neurological outcomes in TBI. 

### 2.6. Cyclosporin

Cyclosporin is a potent immunomodulator whose therapeutic use dates from the early 1980s for prevention of organ transplant rejection; its main mode of action lies in inhibiting the production of cytokines that regulate and activate T lymphocytes, particularly IL-2 [111]. Cyclosporin A acts on mitochondria by inhibiting the permeabilization transition pore—blocking the release of cytochrome c into the cytosol – thus inhibiting apoptosis. This mechanism makes it an interesting candidate treatment for TBI, with a number of animal models suggesting benefit [112,113,114,115,116,117]. 

### 2.7. Oxytocin

Oxytocin is a neuropeptide synthesized by paraventricular neurons of the hypothalamus and excreted by the neurohypophysis; it is known to play a fundamental role during pregnancy by ensuring the tone of the uterus then the initiation of contractions and childbirth, and finally promotes lactation for breastfeeding. Numerous works also suggest its role in social interactions and the pleasure felt during these interactions; in fact, some authors show that mice lacking an oxytocin receptor in the nucleus accumbens exhibit disturbances in social interactions [118]. In addition it seems that oxytocin has a neuroprotective effect by a direct action on the microglia in the context of systemic inflammation (injection of lipopolysaccharide [LPS]), post-traumatic stress, and stroke [119,120,121,122]. All of these arguments make it possible to imagine that a treatment modulating the activity of oxytocinergic neurons, by attenuating microglial activation, could improve the quality of life of patients suffering from cerebral pathologies such as post-traumatic encephalopathy and which are manifested by social interaction disorders [123]. 

### 2.8. Erythropoietin

Erythropoietin (EPO) is a glycoprotein regulating erythropoiesis in the bone narrow; it is naturally produced by kidney. It has also been found in the brain even though it is on the upper limit of the molecular weight threshold to pass the BBB, and may have a neuroprotective role in TBI [124]. TBI leads to an upregulation to EPO receptor expression particularly in neurons, glial, and endothelial cells. EPO could promote neuroprotection in TBI by activating the antiapoptotic cascade JAK-2/NFkB [125] and PI3K, promoting STAT5 homodimerization [126]. Preclinical studies demonstrated that EPO could be antioxidant, antiedematous and also anti-inflammatory [127,128], and that it could reduce cell loss and promote neurogenesis [127,129]. However, in 2015, a double-blind, placebo-controlled trial undertaken in 29 centers in seven countries did not show a beneficial effect of the EPO treatment [130], but in 2020, Katiyar et al. published a meta-analysis including research studies through December 2019 showing that EPO could reduce 6-month mortality (though not in-hospital mortality), neurological outcome, and risk of deep vein thrombosis [131]. These results suggest the need for other clinical trials. 

On the other hand, because of its essential roles during neurodevelopment (genesis, survival and differentiation of neural cells), EPO could also be a very interesting drug for children suffering from TBI. Extended high doses of EPO seem to prevent long term cognitive deficit and white matter loss visible in diffusion tensor imaging in infantile animal controlled cortical impact (CCI) studies [132]. 

EPO is already in use as a treatment for anemia, particularly in patients with renal insufficiency. It is a very low-toxicity treatment and its anti-apoptotic effects could improve the outcome of TBI patients. It is important to underline the interest of this treatment in the perinatal context, notably because of its essential character during neurodevelopment, which makes it a particularly interesting candidate in children.

### 2.9. Others

Other anti-inflammatory drugs targeting the selective activity of certain cytokines have also been tested. These include, for example, anti-TNFα [133]: etanercept. In mouse models, etanercept reduced microglial and astrocytic activation while stimulating neurogenesis, and thus improved post-traumatic cognitive performance [134,135,136]. A rat model of lateral fluid percussion showed attenuation of cerebral ischemia, neurological motor deficits, and numbers of microglia-TNFα double positive cells with etanercept therapy. In humans, the administration of anti-TNFα after a stroke promotes regression of pain and chronic deficits [137,138]; these results are also found in a post-traumatic context with a significant improvement in motor, sensory and cognitive functions [139]. 

Other authors are developing neuroprotective strategies from the IL-1 receptor antagonist (IL-1ra) to which both IL-1β and IL-1α bind. The use of a transgenic mouse hyperexpressing this antagonist makes it possible, by blocking the IL-1 pathway, to decrease the overall production of cytokines and in particular of TNF-α and IL-6 [140]. IL-1ra-treated animals show fewer nitric oxide synthase-2-positive cells in and around the lesion [141] and reduction of oligodendrocyte loss [142]. Anakinra, a recombinant IL1-R antagonist, reduces neuroinflammation and preserves post-TBI cognitive function in mice [143]. Conversely the use of selective antagonist interleukin 1β had no effect on motor recovery [144]. Some authors have studied the effect of anakinra on the mouse eye after blast-mediated traumatic brain injury, where anakinra treatment resulted in a preservation of retinal ganglion cells function and structure compared with saline treated bTBI mice, suggesting that IL-1 blockade also could also prevent axonal damage after blast [145]. Early injection of specific anti-IL-1β antibodies minimizes microglial activation, decreases neutrophilic and T lymphocyte infiltrates and reduce lesion volume [146] and cerebral edema [147]. In humans, a single center phase II randomized therapeutic trial shows the safety of injection of anti-IL-1 as well as its anti-inflammatory action on 20 patients; however, statistically significant clinical improvement was not shown, perhaps in part because the study was underpowered [148]. 

IL-6 also appears to be a robust marker of both neuroinflammation and intracranial hypertension in the setting of TBI [149,150], and injection of anti-IL-6 antibodies reduced the production of pro-inflammatory cerebral cytokines and improved motor functions in mice [151]. 

HMGB1, high mobility group box 1, is one of the Damage-Associated Molecular Patterns (DAMPs) proteins; it is a normally intracellular chromatin protein that is released by necrotic cells [152] and macrophages in response to stimulation by LPS or IL-1β [153]. It is believed to act on TLR receptors and, at the microglial level, it activates the NFkB pathway and the production of superoxide [154]. HMGB1, alarmin early involved in post-traumatic neuroinflammation and involved in neurogenesis, thus appears to be a particularly promising therapeutic target by many approaches. Targeting HMGB1 was shown to reduce microglial activation, the production of pro-inflammatory cytokines, and cerebral edema, and to improve the post-traumatic neurological outcome in mouse models [155,156,157]. In another murine model of CCI, HMGB1 antagonism reversed brain damage, and significantly reduced brain edema by protecting BBB integrity [157]. A treatment with an anti-HMGB1 monoclonal antibody improved post-traumatic motor and cognitive functions for fourteen days after the injury, prevented neuronal hippocampal death, and reduced microglial accumulation [158]. HMGB1 appears to be a therapeutic target, however at present the toxicity of using an HMGB1 antagonist has not been studied. Further work is needed to determine the potential utility and use of this treatment in humans.

## 3. Anesthetic Agents

### 3.1. Halogenated

Halogenated agents are hydrocarbons some parts of which are substituted by a halogen atom (bromine, chlorine or fluorine), thus explaining their name. They are powerful anesthetics first used by Morton in 1846 with ether, then chloroform [159]. It was not until a century later that methoxyflurane was marketed, which was subsequently discontinued due to its renal toxicity. Similarly, halothane and endoflurane are no longer used because of their cardiovascular toxicity. Isoflurane, desflurane, and sevoflurane, marketed in France in 1984, 1990 and 1996 respectively, are the three halogenated agents most used in clinical practice today. Thanks to PET and MRI imaging techniques, halogenated agents have been shown to modify cerebral metabolism, particularly in specific regions such as the thalamus and the reticulate formation [160], thus modifying global neuronal activity. The neuronal effect of halogens is also apparent *in vitro*: they are responsible for a decrease in the release of glutamate [161] with inhibition of the transmission of nerve impulses [162,163] as well as a potentiation of the inhibitory effect of gamma-amino-butyric acid (GABA).

Halogens facilitate neuroprotection by decreasing the brain’s electrical activity and its consumption of oxygen and glucose [164,165]. In a model of stroke, isoflurane has been demonstrated to inhibit microglial activation through the Notch pathway [166]. Isoflurane could also decrease the incidence of brain edema by downregulating aquaporin 4 [167,168]. In a study using controlled cortical impact, adult rats pre-teated with isoflurane presented a better cornu amonnis (CA) 3 neuronal survival and better performance in the Morris water maze and beam walking, thus a better motor coordination and a better memory [169]; these results were confirmed in other studies [170,171]. Sevoflurane may also attenuate inflammation [172,173] without modulating microglial activation [172]. However, in a model of cerebral arterial occlusion in rats, Dang et al. demonstrated that sevoflurane treatment impacts microglial/macrophage dynamics, migration, and phagocytosis—and so, indirectly, microglial activation – and promotes brain repair [174]. Moreover, in a model of neonatal ischemia, sevoflurane was shown to promote neuronal survival through the regulation of PI3K/Akt, and to improve neurocognitive performance [175,176]. Isoflurane shows similar results [177,178], but in an in vitro BBB model and controlled cortical impact study in mice, sevoflurane protected from brain edema better than isoflurane [179]. Statler et al. showed that treatment with isoflurane after focal trauma in rats improved the neurological score as well as the size of the lesion via an inhibition of the decrease in cerebral perfusion, an inhibition of the excitotoxicity of glutamate, and stimulation of GABA type A receptors [180]. 

To conclude, halogenated anesthetics may be neuroprotective from a mechanistic point of view [181], but the literature on its role in traumatic brain injury remains limited, and further studies are needed.

### 3.2. Inert Gas

Xenon is a colorless, odorless gas, and is the principal inert gas studied for therapeutic purposes in TBI thus far because it is used in anesthesia. Xenon is obtained by a complex air separation process, and its cost of production may unfortunately limit its use clinically. However, it does seem to have neuroprotective properties [182], especially in the context of cerebral ischemia [183]. Its action is not thought to be mediated by GABA receptors but rather by inhibition of NMDA receptors [184], thus reducing trauma-related excitotoxicity. In some models of murine TBI, xenon additionally helps reduce microglial activation and neuronal loss, thereby promoting late neurocognitive development [185,186]. In a study published in 2021, Xenon treatment reduced lesion volume, neuronal loss, microglia, reactive astrocytes, and early locomotor deficits [187]. In 2021, Filev et al. studied the effect of Xenon on gene expression in brain tissue in context of TBI rat model and observed lower expression of inflammatory genes like *Irf1* (Interferon Regulatory Factor 1) in the area of damage [188]. Xenon appears to have few side effects: while it causes bradycardia, it has no effect on hemodynamic stability in contrast to other anesthetic agents. To conclude, Xenon appears to act through a variety of pathways, but among the most likely mechanisms—which could explain its protective effects on brain tissue—is the inhibition of NMDA receptors, which become overactive after brain injury. Given the relative safety of xenon and the results of the present study, the researchers hope to be able to quickly study the effectiveness of Xenon in TBI patients.

Argon, the third gas present in air (0.9%), exhibits anesthetic properties in hyperbaric conditions *via* GABA receptors [189]. Its neuroprotective [190] effect is observed on slices of hippocampal brains subjected to ischemic stress [191] or a trauma [192]. The underlying mechanisms are still poorly understood and the increase in the anti-apoptotic protein B-cell lymphoma 2 (BCL2] has been suggested [192]. In a recent study published in 2021, inhaled Argon reduced brain edema and neuroinflammation, and also accelerated sensorimotor and cognitive recovery. However, these results were not replicated in other rodent studies [193]. Its study in humans has been more limited [194]. To conclude, the evidence for Argon’s neuroprotective effects is less certain than for Xenon in the context of TBI.

Regarding inert gases, the literature on human subjects is very limited. The administration of these gases in intensive care requires a respirator with a closed circuit. Therefore, not only has the therapeutic effect of these gases not yet been proven in humans, but their daily use in intensive care is also inconvenient.

### 3.3. Propofol

Propofol is a highly hydrophobic intravenous hypnotic in the form of a lipid emulsion. Like many intravenous anesthetics, propofol has the ability to decrease brain oxygen consumption, decrease glutamate release, and modulate GABA-A receptor activity [195,196,197]. In the context of TBI, the literature on propofol is quite limited aside from demonstrating its antioxidant effects [198]. Indeed, propofol decreases ROS production, pro-inflammatory cytokine levels [199]. In vitro, propofol decreases oxidative stress [200]. In a CCI rat model, propofol reduced brain edema by suppressing aquaporin-4 expression, and was associated with a decrease in IL-1β and TNF-α levels [201]. Propofol may also be neuroprotective by blocking microglial activation through the modulation of nicotinamide adenine dinucleotide phosphate (NADPH) oxidation [202]: in a study published in 2013, propofol decreased expression of inducible nitric oxide synthase, nitric oxide, TNF-α, IL1β, ROS, and NADPH oxidase. Propofol has also been demonstrated to improve neurological TBI outcomes [203]. However in one study propofol limited reparative processes at the acute phase [204]. Interestingly propofol had protective effect on the intestine following TBI by lowering inflammatory markers expression [205]. Propofol is among the anesthetic drugs that could indeed improve the management of TBI patients.

### 3.4. Dexmedetomidine

Demedetomidine is a selective α-2-adrenergic agonist used for sedation during mechanical ventilation. It has already been demonstrated to be neuroprotective in models of ischemia and excitotoxicity. In CCI rat model, dexmedetomidine reduced secondary BBB damages by upregulating tight junction proteins, promoted neurogenesis, and decreased apoptosis, the latter potentially via suppression of NFkB and NOD-like receptor family, pyrin domain containing 3 (NLRP3] inflammasome activation [206]. Other studies show dexmedetomidine protects against apoptosis particularly in hippocampus via upregulation of Hsp70 [207]; it also protects against axonal injury and synaptic degeneration [208]. This neuroprotective effect could be due to the activation of the mTOR pathway [209], or inhibition of microglial activation [210,211,212].

Dexmedetomidine decreases levels of cytokines such as IL-1ß, IL-6, TNF- α, and IL-8 in a mouse model of TBI, but also in patient serum [213]. Recently, Dexmedetomidine has emerged as a potential neuroprotective agent by several mechanisms in TBI. Further work and particularly randomized clinical trials are needed.

### 3.5. Ketamine

Ketamine is an N-methyl-D-aspartate receptor antagonist shown to present anti-inflammatory effects in models of systemic inflammation [214,215] and brain ischemia [216]. The use of ketamine is very controversial because of its potential role in increasing intracranial pressure (ICP), but in 2013 Chang et al. reviewed the studies that had employed ketamine for sedation, and demonstrated no increase in ICP and eventually a neuroprotective effect [217]. For example, in a model of Maramrou’s weight drop model in mice, ketamine administration ameliorated oxidative stress, induced the expression of NRF2 pathway related factors, and ameliorated secondary brain injury including water content, neuronal apoptosis, and neurological deficit [218]. Ketamine has long been thought to be deleterious in neuroinjured patients, but recently several studies have suggested that it may have neuroprotective effects.

## 4. Hormonal Treatments

The role of the hypothalamic-pituitary axis appears to be major in TBI. In humans, hypopituitarism is indeed observed in association with TBI [219]; in a cohort study following 116 adults with TBI for up to 6 months, patients showed a decrease in estradiol, follicle stimulating hormone (FSH), and luteinizing hormone (LH) followed by an increase in stress hormones and impaired cognitive function. Female hormones may thus have a protective effect. These data corroborate those of numerous studies reporting that women with TBI not only have a lower mortality rate but also a better functional recovery than men [220,221,222,223]. 

### 4.1. Estrogens

In many models of brain damage, early administration of sex hormones may have anti-apoptotic, anti-inflammatory, and antioxidant effects, which can speed up repair processes and reduce long-term sequelae [224].

One of the main hypotheses regarding estrogen is based on the increased production of the protein sonic hedgehog, which regulates neuronal differentiation and may promote neuronal regeneration and protection [225]. Estrogens are also believed to have antioxidant properties [226,227,228,229] and to promote astrocytic production of glutamine, a precursor of glutamate, which will be captured by glutamatergic neurons [230,231]. They also act on microglial pro-inflammatory mediators such as the activation of NFkB or inducible NO synthase [232]. Emerson et al. observed in 1993 that the administration of estrogen before TBI improves the fate of male rats and paradoxically worsens that of females [233]. In addition, Kim *et al*. showed a decrease in intra-cranial pressure (ICP) and an increase in cerebral perfusion pressure, partial oxygen pressure, and glycolysis at the lesion [234]. Estrogen seems to have powerful effects on acute brain edema. Furthermore, 17 ß-estradiol treatment, the most abundant and potent endogenous vertebrate estrogen, alleviated neurological deficits, neuronal injuries, brain edema, and pro-inflammatory marker expression such as TLR4, NFkB, IL1-β, IL6 or TNFα in TBI [235]. 17 ß-estradiol protects against programmed cell death in the cortical pericontusional zone by decreasing caspase-3 activation [236]. Estrogens decrease free radical production and oxidative stress after TBI [237]. Bazedoxifen, a third generation selective estrogen receptor modulator, promoted neuroprotection by suppressing the activation of the MAPK/NFkB pathway and attenuated cognitive dysfunctions [238]. In order to better understand if the neuroprotective effect of estrogen was due estrogen receptor alpha or beta, some authors compared the effect of ER alpha and beta agonists: estradiol effect on brain edema, BBB permeability and neurological outcome was mediated through both receptors [239,240]. Estrogens could be an interesting therapeutic for both prevention and intervention post-TBI *via* multiple mechanisms. 

### 4.2. Progesterone

Similar to estrogen, several studies describe a protective role for progesterone [241,242,243]. Three independent studies showed a reduction in post-traumatic brain edema after administration of the hormone in animals [244,245]. One of the explanations lies in the modulation of the expression of aquaporin 4 [246] and the p-glycoprotein. In addition, some authors find a decrease in post-traumatic DAI [247]. Moreover, progesterone presents anti-inflammatory effect reducing cytokine levels like IL1, IL-6, TLR2, TLR4, TNFα, NFkB activity and microglial activation [248,249]. Progesterone protects again lipid peroxidation [250]. Finally, progesterone treatment promotes myelin formation in Schwann cells and increases the number of mature oligodendrocytes [251,252,253].

According to studies from the Wagner laboratory, progesterone plays a crucial role in the development of brain and behavior [254,255]. The progesterone receptor is expressed in the forebrain during brain maturation in the rodent, and may influence neuronal migration, synaptogenesis, and cell death [256,257]. On a pediatric postnatal rat model of weight drop injury, the authors evaluated the effects of progesterone and magnesium separately and administered together: combination therapy was superior to progesterone alone for improving neuronal survival and overall long-term outcome; spatial learning and memory retention at three weeks was improved by each of these treatments alone and in combination [258]. Progesterone decreases anxiety after TBI in the immature brain [259]. Very promising results from clinical trials showed a neurodevelopmental improvement in extremely preterm infants (<1000 g) at 5 years [260,261]. Multicenter randomized trials are needed to confirmed these results. 

In adults humans, progesterone promotes neurocognitive recovery [262,263,264,265,266], but unfortunately these data are controversial, and recently two randomized controlled studies investigating the benefit of treatment with intravenous progesterone in the acute phase of trauma did not show any benefit of this treatment compared to placebo [267,268].

To conclude, the effect of a progesterone treatment differs between children and adults. One of the explanations is that children’s brains are more sensitive to brain edema, so therapies aimed specifically at decreasing cerebral edema may have greater effect in children than in adults. 

## 5. Vitamin Supplementation

### 5.1. Vitamins B

Vitamin B2 (riboflavin) is provided through food (meat and dairy products). Easily absorbed, it is necessary for normal cellular function and has strong antioxidant effects [269]. It delays neuronal death *in vitro* under excitotoxic conditions and in a dose-dependent manner [270]. Despite its potent antioxidant status, there have been very few studies on neuroprotection with riboflavin. In rat TBI models, it reduces edema, lesion size, and astrocyte activation, and facilitates cognitive and sensorimotor recovery [271,272]. B2 treatment also reduces behavioural troubles in the bilateral tactile removal test and improves reference and working memory [271].

Vitamin B3 (nicotinamide) is the amide form of nicotinic acid. Its neuroprotective action is widely characterized after TBI and stroke [273]. Its effects are manifold and include energy supplementation, scavenging of free radicals, and reduction of cell activation, apoptosis, and DNA damage [274]. The combination of these mechanisms makes nicotinamide an attractive candidate for the treatment of brain damage. In vivo, vitamin B3 administration is effective in several models of traumatic injury: it improves sensory, motor, and cognitive functions after frontal injury [275,276,277] and unilateral injury [278,279,280]. Acutely (<7 days after injury), vitamin B3 treatment reduces apoptosis, neuronal degeneration, edema, and BBB damage; finally, it decreases astrocyte activation and lesion volume [281], the latter even after a substantial latency period [278,279,282]. 

Preclinical evidence in rats suggests that nicotinamide may be an interesting treatment to explore in a clinical population. However, the putative neuroprotective dose in humans could be responsible for toxicity not yet evaluated in the literature. However, even taking into account possible toxicity problems, nicotinamide could exert protective effects in post-trauma and be particularly interesting to use in combination therapies because it is relatively easy to administer with few negative drug interactions.

Vitamin B6 (pyridoxine) is a water soluble vitamin, easily metabolized and excreted [283]. Pyridoxal 5′-phosphate (PLP) is the active coenzyme of vitamin B6 and is essential for the metabolism, catabolism, and transamination of amino acids [284]; according to some studies, it may have a neuroprotective effect by promoting glycogenolysis and reducing excitotoxicity [285,286]. Vitamin B6 reduces tissue damage post-TBI [287]. However, chronic high doses of vitamin B6 can lead to considerable neuronal toxicity with behavioral disturbances and problems with balance and walking [288,289], which limits the feasibility of long-term treatment at high doses. 

Vitamin B9 (folic acid) is well known for its role in neural tube closure but also in the process of cell division, DNA synthesis, and maintenance of DNA methylation [290]. The literature is quite limited concerning TBI and does not permit conclusions regarding a therapeutic effect of vitamin B9: if certain authors conclude folic acid improves cognitive function in a pig model [291], such results were not found in a mouse model [292]. In a piglet pediatric model of TBI, piglet treatment with folic acid presented better exploratory interest and locomotion on day one, but not after. Axonal injury was not affected by the treatment.

### 5.2. Vitamin C

Ascorbic acid (AA), or vitamin C is widely recognized as one of the most important endogenous free radical scavengers [293]. It has also been suspected to have neuroprotective action by decreasing excitotoxicity-induced damage [294]. As part of the general metabolic dysfunction post-TBI, tissue levels of ascorbic acid have been shown to be immediately greatly reduced [295] and do not return to normal until 72 h after [296]. Polidori et al. published a cohort study of TBI patients in 2001, showing decreased ascorbic acid plasma levels in TBI patients compared with healthy controls, and this decrease was inversely correlated with the severity and the neurological outcome [297]. In addition, reduced levels of vitamin C are associated with an increase in lesion volume [298]. Despite the apparent disruption of vitamin C function in TBI, relatively few studies in humans have attempted direct vitamin C supplementation. Two studies have shown that treatment with vitamin C preserves ascorbic acid at appropriate levels in rat models of TBI, and maintained the production of superoxide dismutase with improved motor function in the course of the trauma [299]. Combined with vitamin E, ascorbic acid could improve cellular defense mechanisms and protect against oxidative effects of TBI [300]. In addition to its free radical scavenger activity, vitamin C may promote the integrity of the BBB by regulating the balance between metalloproteinase 9 (MMP-9) and free radical scavengers like Nrf2 [301,302]. These results warrant further study. Prophylactic ascorbic acid 2-glucoside significantly limited TBI-induced oxidative stress and mitigated motor dysfunction while the effects of therapeutic treatment were limited [303]. Clinically, the effect of vitamin C on critically ill patients is not particularly beneficial [304]. Literature on ascorbic acid treatment in TBI patients is generally consistent with this finding but inherently limited. The only randomized controlled trial on high-dose vitamin C administration for TBI was conducted in 2011 with 23 patients, but the authors did not observe any improvement in outcomes [305]. With its high safety, low cost profile, however, as well as the antioxidant properties above with potential to target TBI mechanisms, ascorbic acid remains a promising candidate for the acute stage of TBI management, e.g., in prehospital administration [306]. 

### 5.3. Vitamin D

A fat-soluble vitamin found in food, vitamin D is a secosteroid associated with peripherical calcium homeostasis. It is synthesized by the human body from a derivative of cholesterol and converted to its active form via ultraviolet radiation from the sun. Much of what is known about the neuroprotective effects of vitamin D comes from data on vitamin D deficiency [307] which suggest that it modulates apoptosis and reduces oxidative stress, inflammation, and excitotoxicity.

In the context of TBI, vitamin D was initially explored with progesterone [308] for its potential to act synergistically, as well as to study the relationship between age-related vitamin D decline and brain damage [309]. Other work not only observes an improvement in cognitive function [310] but also a reduction in inflammation and neuronal loss [311]. Although effective in adult rats, it appears that this combination may be most beneficial in middle-aged animals, potentially due to greater existing vitamin D deficiencies in that subpopulation. In older animals, this combination significantly reduces astrocyte proliferation and reduces neuronal loss [312]. The reason for the synergy of vitamin D and progesterone has not yet been fully elucidated, but a study suggests that it is the combination of a decrease in astrocyte activation and phosphorylation of NFκB [311].

Yang et al. demonstrated in 2021 that vitamin D supplementation reduces brain edema and inflammation while improving BBB integrity and behavioral function post-TBI [313].

Clinically, vitamin D supplementations in mild to moderate TBI patients at the acute phase of injury may improve long-term performance and cognitive outcomes evaluated with the Mini-Mental Status examination and the GOSE [314]. 

In 2020, thirty-five patients with moderate TBI were randomly allocated to a one-time oral dose of vitamin D; an improvement in level of consciousness was observed after 7 days in the vitamin D treatment group [315], confirming results of a previous study. Although further studies are necessary to validate the synergistic effects of vitamin D with progesterone, there is increasing evidence that this combination is an interesting therapeutic strategy and unlikely to exhibit toxicity. However, further exploration of the effects in younger animals, a better understanding of the therapeutic window, and more robust characterizations of functional recovery must be established before proceeding with further clinical trials.

### 5.4. Vitamin E

Vitamin E is a fat-soluble vitamin comprising a set of eight molecules, the most active biological form of which is alpha-tocopherol. It acts as an antioxidant against oxygen derivatives and in particular those resulting from the oxidation of fatty acids [316]. In combination with polyethylene glycol, it reduced mortality by 50% in TBI models and improved motor function [317]. Similar results were seen on neurocognitive function with alpha tocopherol alone given up to 90 days after trauma [318,319]. Alpha tocopherol reduced microscopic brain damage and also promoted nerve regeneration probably by reducing Nogo-A and NgR expression [320]. Although these behavioral effects are important, a study showed limited efficacy of vitamin E on lipid peroxidation in the acute post-injury phase [321]; others have demonstrated a later improvement in markers of oxidative stress. Vitamin E could also present neuroprotective effects for TBI associated dementia [322]. However, despite its high fat solubility and low toxicity, it takes a considerable time to reach effective concentrations in the central nervous system [323] and at these doses can be responsible for bleeding.

## 6. Ions

### 6.1. Magnésium

Over the past decades, many studies have suggested the interest of magnesium (Mg^2+^) in post-TBI recovery through improved cognitive functions [324]. The efficacy of Mg^2+^ therapy in promoting functional recovery across a variety of animal models of TBI is well demonstrated [325,326,327]. Administration of Mg^2+^ in animals with a normal diet not only improves sensorimotor function but also reduces certain histological damage such as rupture of the BBB, cerebral edema, neuronal death [328], apoptosis [329] glial proliferation [330,331,332,333] and brain damage [334]. Magnesium pre-treatment prevented injury impairments in working and reference memory via hippocampal ERK activation, and neuronal loss. At 1 week post-injury, magnesium treatment improved posttraumatic anxiety and depression [335]; in another study, four weeks after magnesium treatment improved also sensorimotor performance and recovery [336]. We have to note that some authors tried to compare magnesium sulfate and magnesium gluconate and they conclude that both have neuroprotective effects post-TBI [337], although there have been recent clinical trial failures for both TBI and stroke [338,339]. In 2017, Natale et al. showed that magnesium sulfate administration did not modify mean arterial pressure or alter cardiac conduction [340] in children population. 

### 6.2. Zinc

Zinc plays a controversial role in the pathophysiology of TBI [341]. Many studies have identified increased and toxic levels of zinc after an experimental TBI while others have highlighted zinc deficiency to be deleterious and have shown zinc supplementation to be an effective therapy. Moreover recent studies showed that TBI did not link to drastic change of brain zinc level [342]. Several studies nevertheless link the accumulation of post-traumatic zinc to neuronal cell death by excitotoxicity [343,344] that could be due to rapid shifts in zinc localization [343,344]. The zinc mechanisms of toxicity is not well elucidated but it seems that like microglial dysfunction, it could induce reactive oxygen species [345] leading to mitochondrial disruption and neuroinflammation [346]. Other hypotheses are raised in the literature like mitochondrial dysfunction [347] or poly (ADP-ribose) polymerase (PARP) activation [348]. Mice without vesicular zinc presented more damages after TBI compared to wild type controls [349]. Zinc supplementation could also promote neurogenesis [350].Thus, the elimination of excess zinc, by chelation for example, is evaluated through several studies with a mixture of beneficial results [351], no effect [350] or harm (Need ref for harm).

Because the observation of TBI patients shows zinc deficiency [352], zinc supplementation was evaluated in patients and rats and indeed shows an improvement in cognitive and motor functions [353,354]. In a rat fluid percussion model, intraventricular injection of Ca EDTA provided neuroprotection in the CA1 region of the hippocampus, and dentate [343] and upregulated neuroprotective genes coding for heat shock proteins 27 and 70, and anti-apoptotic protein p21 [351]. Nonetheless one study demonstrated that zinc chelation participated to neuronal damages when hippocampus neurons were overexcited [355]. In addition, zinc chelation did not improve neurological outcome after TBI and increased the pro-apoptotic proteins BAX and caspase 3 two weeks after the injury leading to a second wave of injury weeks after the initial trauma [356]. On the other hand zinc deficiency was associated with ateration in matrix metalloproteinases, BBB disruption, inflammation and angiogenesis [357] but did not worsen behavioral outcome. 

In a model of controlled cortical impact to the medial frontal cortex, rats were fed with a zinc adequate or a zinc supplemented diet: none of these treatment reduced anxiety behaviors but the dietary zinc supplementation improved learning and memory and the combination of intraperitoneal zinc and zinc dietary supplementation was necessary to reduce depression-like behaviors [358]. In another study, chronic zinc supplementation provided behavioral resiliency. Clinically zinc supplementation treatment for three months improved the Glasgow Coma Scale at 2 weeks and 3 months [354]. More recently a double-blind controlled study confirmed this results: zinc supplemented patients had a higher Sequential Organ Failure Assessment and Glasgow Coma Scale [359].

## 7. Omega-3

Omega-3 fatty acids are polyunsaturated fatty acids found in fatty fish and in certain plants; they have received a lot of attention, particularly in the prevention of cancer and cardiovascular risk factors. Regarding the CNS, they play multiple protective roles: both antioxidants, anti-inflammatories, and modulators of neurotransmission [360]. They have been the subject of numerous works in a traumatic context [361,362]. They have multiple modes of action: first of all, they reduce neuronal death by increasing the level of BDNF [363,364], then, they are powerful anti-inflammatory agents capable of lowering the level of cytokines such as TNF-α, IL-6 or reactive C protein [365], and finally they have other more controversial effects such as a decrease in excitotoxicity via the modulation of AMPA receptors [366,367]. While the biochemical evidence is quite promising, there are relatively few studies that examine the functional results associated with omega-3 acids and TBI. Some studies show that the depletion of omega-3 acid reserves leads to worsening motor and memory deficits [368], conversely, others show that supplementation before an injury leads to an improvement in motor and learning capacity [369,370], preserved the integrity of myelinand maintained post-TBI conductivity [370]. Dietary supplementation with docosahexaenoic acid reduced brain injury (measure with marker of cellular injury and apoptosis like APP and caspase-3), inflammation (CD68 positive cells) and improved memory assessment [371]. Omega-3 polyunsaturated fatty acids treatment promoted a shift from microglial pro-inflammatory phenotype to anti-inflammatory on, reducing inflammatory factors level probably mediated by decreasing HMGB1-medication activation of the NFkB pathway [372]. Recently some authors showed that pretreatment with omega-3 in TBI mice improved glymphatic clearance and suppressed expression of aquaporin 4 [373]. The accumulation of evidence in favor of the neuroprotective effect of omega-3 fatty [374] acids is quite promising in the setting of TBI but further study is still needed.

## 8. Discussion

Traumatic brain injury remains a significant health public problem and the leading cause of disability in patients under 40 years old. Post-traumatic encephalopathy can present with a broad range of symptoms, including somatic, neuropsychiatric, motor, and cognitive disorders, with lifelong consequences. These long-term consequences have been the target of clinical research for the last ten decades. In spite of a recent better understanding of the underlying neuroinflammatory mechanisms, there is currently no treatment proven to reduce post-traumatic handicap. In this review we have aimed to establish a list of current therapeutic drugs tested in both animal models of TBI and in humans, including anti-inflammatory medications such as glucocorticoids and NSAIDs, more specialized anti-inflammatory treatments, anesthetic agents, hormonal treatments, vitamins and ions (magnesium, zinc), and stem cell therapies. The problem remains that currently none of these drugs have been demonstrated to convincingly improve TBI patient outcomes, and therefore none is routinely used for TBI patients. 

Even if the mechanisms responsible for the progression of secondary lesions to tertiary lesions are not well elucidated, the best post-traumatic time period for intervention seems to be the acute phase; later, injuries become established. Treatment during intensive care unit hospitalization is therefore a key element of managing these lesions; for maximum efficacy, it should be administered too late: the earlier the better. As the goal of treatment is to block the immune response before it gets out of control and creates irreversible damage, the best window of opportunity is during hospitalization in intensive care or just before. Once the lesions are already constituted, treatment seems without benefit. However, the ICU resuscitation period is also a risky period for use of anti-inflammatory treatment, since the patient is extremely fragile and at increased risk of opportunistic infections. Hence the importance of choosing a treatment that controls brain inflammation without weakening the patient’s immune defenses against infections. 

The difficulty lies mainly in the heterogeneity of the brain damage; it is indeed a multifactorial pathology involving several cell types, mainly neurons, but also the brain’s own immune system (microglia, astrocytes) and the peripheral immune system, which communicate with each other owing to the hyperpermeability of the blood-brain barrier. As it seems unlikely that a single drug could effectively act on all the actors involved in the inflammatory response, one solution could be to use a combination of several treatments with different targets and modes of action. One of the most studied cellular targets is microglia. Indeed, some studies on other models of brain inflammation show that blocking microglial activation is sufficient to prevent brain damage, particularly white matter damage [375]. We can therefore imagine a therapeutic strategy that targets only microglia and which could improve TBI patients’ prognosis.

Aside from consideration of medical intervention, rehabilitation also seems to be crucial. Early intervention produces better outcomes: Andelic et al. showed that patients with early rehabilitation training had higher GOSE and Disability Rating Scale (DRS) at 12 months. Several technologies have been developed recently [376], including transcranial magnetic stimulation (TMS) and transcranial direct current stimulation (tDCS). These procedures are painless, noninvasive, and without major adverse effects. For example, in a case study, throughout five courses of transcranial magnetic stimulation, tinnitus symptoms improved [377]. In a double-blind randomized controlled trial published in 2015, repetitive TMS showed significant effect on depression symptoms and cognitive function [378]. These results were confirmed by two reviews [379,380], which found TMS to improve depression and anhedonia symptoms, auditory hallucinations, tinnitus, and autonomy in activities of daily living. 

Among other novel rehabilitation techniques, some studies have highlighted the potential of virtual reality training. Computer-aided training can stimulate auditory, visual, and cognitive functions such as memory, attention, concentration, and proprioception. The recreational and game-like features of this technique promote participation and patient adherence to treatment. Such computer-based cognitive rehabilitation can improve the use of compensatory strategies, enhance memory, and alleviate post-traumatic mood disorders [381,382]. Another kinesthetic, multisensory rehabilitation method described recently is music therapy [383], shown to stabilize emotions [384] and promote enhancement of executive functions [385]. And finally, functional electrical stimulation, a low-frequency pulse current, can promote functional reconstruction, with task-oriented functional electrical stimulation demonstrating particular benefit in patients with hemiplegia. Social support—the belief that one is cared for and loved, that one belongs to a network of communication and mutual obligations—plays an essential role in recovery as well. Sinnakaruppan et al. showed in 2005 that caregiver and family member education programs help to relieve stress and anxiety and to promote recovery [386]. 

The main limitation of this review is that it is not exhaustive or systematic. It allows discussion and citation of the major publications in the field and for the main drugs tested in head trauma. Despite the importance of the literature on the subject, none of the treatments cited in this review has been proven to be neuroprotective and therefore cannot be recommended. The literature is still burgeoning and many studies are currently underway in humans to evaluate some of these strategies. 

Several studies suspect the role of neuroinflammation and in particular microglial activation in the generation of tertiary post-traumatic lesions, and therefore in longer-term post-traumatic disability.

**Table 1:** Principal therapeutic drugs for traumatic brain injury and their mode of action.

	Drugs	Mode of Action										Pediatric Studies
		Inflammation	Microglial/ Astrocytic Activation	Excitotoxicity	Anti-Oxydative	Apoptose	Œdema	Mitochondria	Neuronal Death/Neurogenesis	Cerebral Metabolism	BBB	
Antiinflammatory drugs	GC					[387]	[44,50]					
	NSAI	[56]										
	Statins	[70,71,72]	[64]	[388]		[59,60]	[389]		[61,62,66]		[389]	
	Melatonine	[390]	[390]		[78]			[86,391,392,393]			[394]	[91]
	Minocycline	[97,395]	[97,110]				[396]					[395,397,398]
	Ciclosporin					[113]	[399]	[112,115,116,117]				
	Oxytocin											
	Anti-TNF-α	[136,137,139]	[136]				[400]		[135]			
	Anti-IL1	[140]	[401]									
	Anti-IL-1β	[146]	[146]				[147]					
	Anti IL-6	[151]										
	Anti-HMGB1	[156,402]	[155]				[156,157]					[402,403,404,405,406]
Anesthesic agents	Hallogenous											
	Argon	[194]										
	Xenon	[186,407]	[183]						[186]			
	Propofol	[199,203]	[202]		[408,409]					[198]		
Hormons	Œstrogenes		[231,232]		[228,229]	[227]			[227]			
	Progesterone	[262]			[242,262]		[244,245,246]	[403]				
Vitaminic supplementation	Vitamin B2		[271]		[269]		[271]					
	Vitamin B3			[281,410]	[281,410]	[281]	[410]				[281]	[411]
	Vitamin B6			[284,286]	[284,286]							
	Vitamin B9											[291]
	Vitamin C			[294]	[293]							
	Vitamin D	[311]		[311]	[312]	[412]						
	Vitamin E				[318]		[413]					
Ions	Magnesium					[332]	[331,333]				[331]	
	Zinc				[414]							
	Omega-3	[365]		[366,367]								

BBB: blood brain barrier; IL: interleukine; NSAI: non-steroid anti-inflammatory.

## 9. Conclusions

Although there is currently no specific immunologically-targeted treatment for adult or pediatric head trauma, this review shows the substantial number of preclinical and clinical trials conducted to date. The only recommendations are those involving the prevention of secondary injury and treatment of intracranial hypertension. This multimodal and multidisciplinary management has effectively improved TBI mortality while increasing the rate of late disability. It therefore seems essential to implement and enrich research in this field.
